# Supplementary material for: Predicting visual function by interpreting a neuronal wiring diagram
Source: Nature. 2024 Oct 2;634(8032):113–23. doi: 10.1038/s41586-024-07953-5 (PMC11446822; doi:10.1038/s41586-024-07953-5)
Supplement: Supplementary file 6 — CRF and ERF predictions for individual TmY4 and TmY9 cells. Analogous to Supplementary Data 3, but for TmY target types. Shown are the top four monosynaptic pathways, the strongest pathway passing through each of the top ten intermediary types (ranking from Extended Data Fig. 7), and the trisynaptic pathway Tm1–TmY–Dm3–TmY (see the section entitled Prediction of spatial normalization). [file 41586_2024_7953_MOESM6_ESM.zip › DataS4/TmY9q/720575940627032337.pdf]

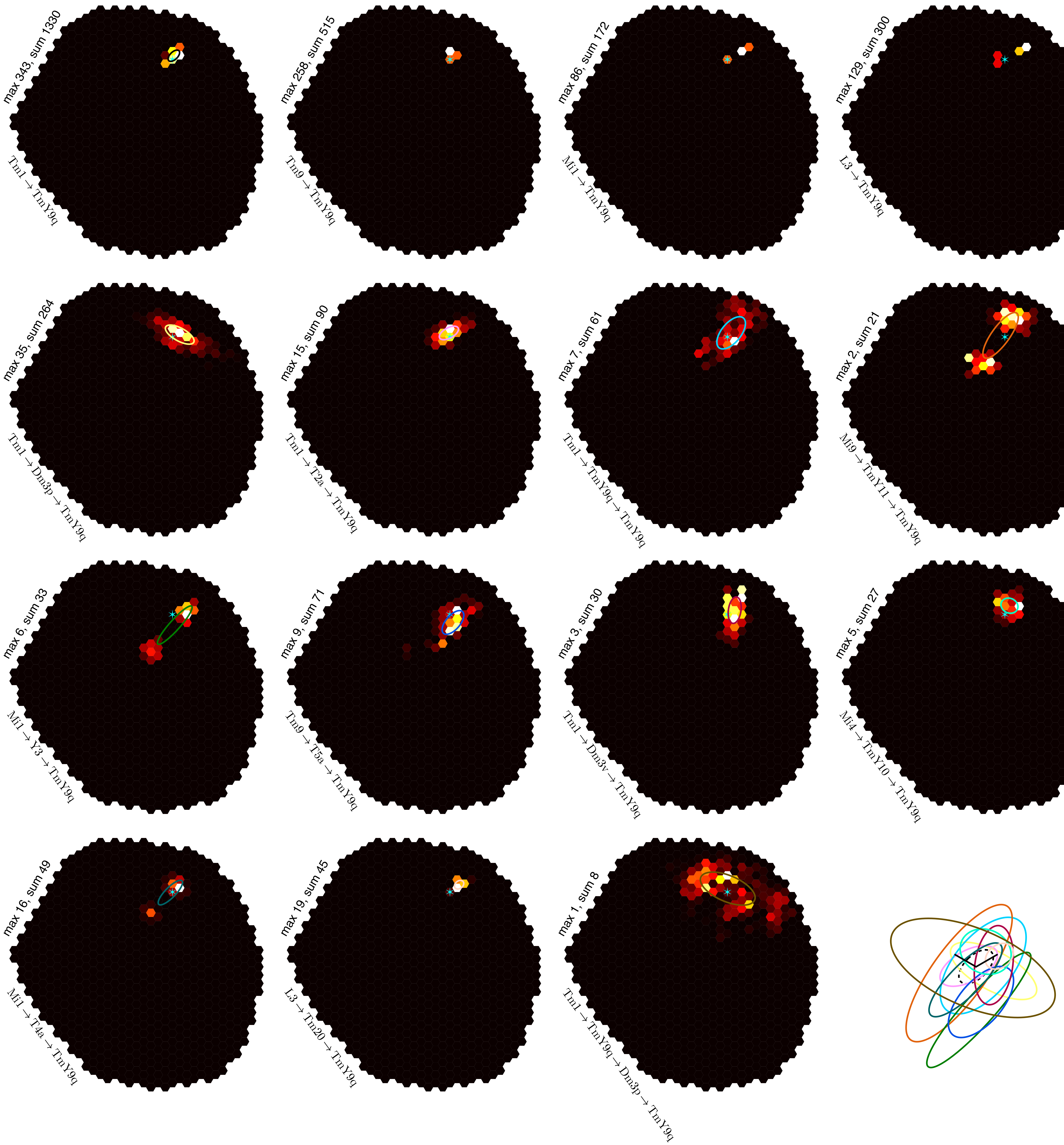
max 343, sum 1330
Tm1 → TmY9q
max 258, sum 515
Tm9 → TmY9q
max 86, sum 172
Mi1 → TmY9q
max 129, sum 300
L3 → TmY9q
max 35, sum 264
Tm1 → Dm3p → TmY9q
max 15, sum 90
Tm1 → T2a → TmY9q
max 7, sum 61
Tm1 → TmY9q → TmY9q
max 2, sum 21
Mi9 → TmY11 → TmY9q
max 6, sum 33
Mi1 → Y3 → TmY9q
max 9, sum 71
Tm9 → T5a → TmY9q
max 3, sum 30
Tm1 → Dm3v → TmY9q
max 5, sum 27
Mi4 → TmY10 → TmY9q
max 16, sum 49
Mi1 → T4a → TmY9q
max 19, sum 45
L3 → Tm20 → TmY9q
max 1, sum 8
Tm1 → TmY9q → Dm3p → TmY9q
